# Supplementary material for: Uptake of appointment spacing model of care and associated factors among stable adult HIV clients on antiretroviral treatment Northwest Ethiopia
Source: PLoS One. 2022 Dec 30;17(12):e0279760. doi: 10.1371/journal.pone.0279760 (PMC9803219; doi:10.1371/journal.pone.0279760)
Supplement: Attachment — Submitted filename: Response to Reveiwers.docx [file pone.0279760.s002.docx]

**Response to Reviewers**

We are happy for your constructive comments and suggestions. We are also learned a lot from these substantial scientific comments to improve the quality of this manuscript. We are addressed your comments and suggestions as stated below. The manuscript was revised as indicated in the highlights and track change.

Journal Requirements:

When submitting your revision, we need you to address these additional requirements.

1. Please ensure that your manuscript meets PLOS ONE's style requirements, including those for file naming. The PLOS ONE style templates can be found at <https://journals.plos.org/plosone/s/file?id=wjVg/PLOSOne_formatting_sample_main_body.pdf> and

<https://journals.plos.org/plosone/s/file?id=ba62/PLOSOne_formatting_sample_title_authors_affiliations.pdf>

Please review your reference list to ensure that it is complete and correct. If you have cited papers that have been retracted, please include the rationale for doing so in the manuscript text, or remove these references and replace them with relevant current references. Any changes to the reference list should be mentioned in the rebuttal letter that accompanies your revised manuscript. If you need to cite a retracted article, indicate the article’s retracted status in the References list and also include a citation and full reference for the retraction notice.

**Tank you!** We are accepted your comment. We are prepared according to the journal guidleine and checked all refernces. We found only one “Cawley C, Nicholas S, Szumilin E, Perry S, Quiles IA, Masiku C, Wringe A: **Six-monthly appointments as a strategy for stable antiretroviral therapy patients: evidence of its effectiveness from 7 years of experience in a Medecins Sans Frontieres supported programme in Chiradzulu district, Malawi**. In: *Journal of the International AIDS Society: 2016*: John Wiley & Sons Ltd the Atrium, Southern Gate, Chichester, 2016” is identifed as retracted and removed from this list.

**Comments to the Author**

1. Is the manuscript technically sound, and do the data support the conclusions?

The manuscript must describe a technically sound piece of scientific research with data that supports the conclusions. Experiments must have been conducted rigorously, with appropriate controls, replication, and sample sizes. The conclusions must be drawn appropriately based on the data presented.

Reviewer #1: Yes

**Thank you!**

2. Has the statistical analysis been performed appropriately and rigorously?

Reviewer #1: Yes

**Thank you!**

3. Have the authors made all data underlying the findings in their manuscript fully available?

The [PLOS Data policy](http://www.plosone.org/static/policies.action#sharing) requires authors to make all data underlying the findings described in their manuscript fully available without restriction, with rare exception (please refer to the Data Availability Statement in the manuscript PDF file). The data should be provided as part of the manuscript or its supporting information, or deposited to a public repository. For example, in addition to summary statistics, the data points behind means, medians and variance measures should be available. If there are restrictions on publicly sharing data—e.g. participant privacy or use of data from a third party—those must be specified.

Reviewer #1: Yes

**Thank you!**

4. Is the manuscript presented in an intelligible fashion and written in Standard English?

PLOS ONE does not copyedit accepted manuscripts, so the language in submitted articles must be clear, correct, and unambiguous. Any typographical or grammatical errors should be corrected at revision, so please note any specific errors here.

Reviewer #1: Yes

**Thank you!**

5. Review Comments to the Author

Please use the space provided to explain your answers to the questions above. You may also include additional comments for the author, including concerns about dual publication, research ethics, or publication ethics. (Please upload your review as an attachment if it exceeds 20,000 characters)

**Reviewer #1:** This is an important study about uptake of ART. The manuscript is well-written and has a nice layout, which make it easy to read and follow. However, I have a few concerns, which if considered and applied by the authors might improve its readability.

1. It’s unclear how appointment spacing model solves the challenges in line #53-57, it would be helpful for the authors to expound on this relationship in the text.

**Thank you!** We are accepted your constructive comments and included your comment in the document page 3 lines 58-65.

2. Related to the above comment #1, the authors state that ASM is associated with lower probability of death and use citation #7 to back that assertion, but that citation did not have anything to do with that finding. What is the relevance of this citation to this text? Given that observation, it now becomes unclear in the text, what is the benefit of ASM to ART, and why it should be advocated for people on ART. The authors need to cite an original source that developed the ASM model and the study that showed its benefits to people on ART. That will form the foundation and validity of this current study/investigation.

**Thank you!** We are accepted your scientific comment and corrected as your comment in page 3-4 lines 58- 78.

3. How were the three out of the 10 hospitals and nine out of 29 health centers selected for the study?

**Thank you!** In this study, 30% and above health facilities providing ART service to ensure the representativeness and select high case load health facilities served more than 500 HIV patients in East Gojjam Zone were included.

4. On line #91 the authors reported that “high caseload ART facilities which hosted more than 500 cases selected for this study”, but on line #97 they reported that “final sample of 423”.

**Thank you!** All health facilities included in this study, >500 HIV patients enrolled and received ART. The sample size calculated for this study was 423 and this sampled study participants were proportionally allocated to each selected health facilities among stable adult ART patients. We are included the schematic presentation of sampling procedure (figure 1) in the document.

5. The tables have n=415, the authors should consider revising the results section and the rest of the manuscript to reflect that sample size. The number of study participants should be consistent across the manuscript. Right now it is a little bit confusing as indicated above.

**Thank you!** In this study, 415 ART patients were participated in this study with 98% response rate. The remaining study participant were not welling to complete all the questionnaire components resulting 8 study participants were incomplete data and not included in the analysis.

6. PLOS authors have the option to publish the peer review history of their article ([what does this mean?](https://journals.plos.org/plosone/s/editorial-and-peer-review-process#loc-peer-review-history)). If published, this will include your full peer review and any attached files.

If you choose “no”, your identity will remain anonymous but your review may still be made public.

**Do you want your identity to be public for this peer review?** For information about this choice, including consent withdrawal, please see our [Privacy Policy](https://www.plos.org/privacy-policy).

Reviewer #1: **Yes:** Chris B. Agala

**Thank you for your constructive comments and suggestions‼!**
